# A case report of acute claudication due to vena cava thromboembolism in a dog naturally infected with *Leishmania Infantum*

**DOI:** 10.1007/s11259-024-10370-8

**Published:** 2024-04-17

**Authors:** Natalia Calvo-Sánchez, Álex Gómez, Estela Pérez, María Borobia, Lluís Luján, Antonio Fernández, Sergio Villanueva-Saz, Maite Verde, Diana Marteles

**Affiliations:** 1 Department of Animal Pathology, Veterinary Faculty, University of Zaragoza, Zaragoza, 50013 Spain; 2 Clinical Immunology Laboratory, Veterinary Faculty, University of Zaragoza, Zaragoza, 50013 Spain; 3 Instituto Agroalimentario de Aragón-IA2 (Universidad de Zaragoza-CITA), Zaragoza, 50013 Spain; 4 Veterinary Faculty, Veterinary Teaching Hospital, University of Zaragoza, Zaragoza, 50013 Spain

**Keywords:** CanL, Claudication, Dog, *Leishmania Infantum*, Thrombus

## Abstract

Canine leishmaniosis is a vector-borne disease caused by *Leishmania infantum*, and clinical manifestations of infection range from absent or severe to fatal and result from immune-mediated mechanisms. In dogs, the most common clinical signs of leishmaniosis include skin lesions and lymphadenomegaly. However, the presence of other nontypical signs has been described, and diagnosing these cases can be challenging. The aim of the present short communication was to describe the impact of the formation of circulating immunocomplexes due to *L. infantum* in a dog with leishmaniosis affected by a massive venous thrombus of the caudal vena cava and external iliac veins. On admission, the dog presented bilateral cutaneous vasculopathy of the thigh and renal disease due to *L. infantum* infection. Two weeks after starting anti-*Leishmania* treatment based on meglumine antimoniate and allopurinol administration, the animal developed acute claudication of the hind limbs with the presence of a thrombus in the caudal vena cava and the external iliac veins and a high level of circulating immunocomplexes detected by enzyme-linked immunosorbent assay. Exacerbation of the humoral immune response, along with deposition of circulating immune complexes in the tissues and the concurrent presence of kidney and liver damage, might have contributed to an imbalance in haemostasis in this patient. Future studies should evaluate and analyse the pathological mechanisms contributing to thrombosis in dogs with leishmaniosis.

**Supplementary Information:**

The online version contains supplementary material available at 10.1007/s11259-024-10370-8.

## Introduction

Canine leishmaniosis (CanL) is a vector-borne disease caused by the protozoan *Leishmania infantum* in European Mediterranean countries. In dogs, clinicopathological manifestations of *Leishmania* infection range from absent to severe manifestations and are fatal in some cases (Papadogiannakis and Koutinas 2015). The most common clinical signs of leishmaniosis include lymphadenomegaly and cutaneous lesions, followed by anorexia, ocular signs, mucous membrane ulceration, and fever, among others (Solano-Gallego et al. 2011; Morales-Yuste et al. 2022). The most frequent laboratory findings observed in CanL patients are nonregenerative anaemia, lymphopaenia, leukocytosis, and a low serum albumin: globulin ratio. Moreover, depending on the affected tissue, biochemical parameters can be altered (Meléndez-Lazo et al. 2018).

Leishmaniosis can be diagnosed in animals, including dogs, cats and ferrets, by different confirmatory techniques (Solano-Gallego et al. 2011; Pennisi et al. 2015; Villanueva-Saz et al. 2021). There are several methods based on direct observation of *L. infantum* amastigotes by cytology, histopathology and immunohistochemistry (Paltrinieri et al. 2016). Molecular techniques for detecting *L. infantum* nucleic acids by polymerase chain reaction (PCR), such as conventional PCR, nested PCR and quantitative PCR (qPCR), are also available. In contrast, enzyme-linked immunosorbent assay (ELISA), indirect immunofluorescence antibody test, and immunochromatographic rapid test approaches represent the most common methods used for detecting specific humoral immune responses against *L. infantum* in infected dogs (Solano-Gallego et al. 2011; Maia and Campino 2018).

Atypical lesions affecting tissues of the cardiovascular, respiratory and musculo-skeletal systems or other uncommon organs have been sporadically reported in CanL, inducing uncommon clinical manifestations and complicating diagnosis (Blavier et al. 2001; Peris et al. 2022).

CanL syndrome has been associated with various haemodynamic alterations, encompassing both haemorrhagic and thrombotic disorders (Petanides et al. 2008; Solano-Gallego et al. 2011). The occurrence of epistaxis in sick dogs can be attributed to a combination of different pathological mechanisms, including haemostatic dysfunction, hypergammaglobulinaemia, and chronic rhinitis. Furthermore, concurrent infections can exacerbate the severity of epistaxis (Petanides et al. 2008). Thrombus formation due to *L. infantum* in dogs is an exceedingly rare phenomenon that has been documented only two times in the scientific literature (Font and Closa 1997; Félix et al. 2008). In both cases, the authors detailed the progression of kidney damage and nephrotic syndrome involving the urinary excretion of antithrombin III. This plasma glycoprotein, synthesized in the liver, functions as an anticoagulant that influencesing various elements within the intrinsic, extrinsic, and common coagulation pathways (Warren et al. 2001). Substantial loss of antithrombin III and albumin through urine can induce a hypercoagulable state, elevating the risk of venous and arterial thromboembolism. Renal vein involvement emerges as the most common manifestation (Font and Closa 1997; Singhal and Brimble 2006).

This case report describes an atypical clinical presentation of CanL consisting of acute posterior claudication due to a massive thrombus occluding the vascular lumen of the caudal vena cava and external iliac veins in a sick dog naturally infected with *L. infantum.*

## Patient history

A 4-year-old spayed female German Shepherd crossbreed was admitted to Veterinary Hospital, University of Veterinary Medicine, Zaragoza (Spain), with a history of exfoliative dermatitis and anorexia. One year prior, the animal was diagnosed with clinical leishmaniosis with hyperglobulinemia and polyclonal gammopathy. Other biochemical parameters were within reference ranges. Urinalysis showed no abnormalities. Diagnosis of the disease in the dog was based on integration of the clinical picture and laboratory abnormalities detected in laboratory tests performed together with a high positive result by serological quantitative ELISA. The dog was classified as a sick dog (D) (Oliva et al. 2010). The patient was treated with meglumine antimoniate at 40 mg/kg BID subcutaneously for five weeks and allopurinol at 10 mg/kg BID PO for one year. Following the meglumine antimoniate treatment period, bimonthly follow-up visits were conducted. Two months after completing the meglumine antimoniate treatment, a follow-up visit to the attending veterinarian was made. At this follow-up visit, a physical examination and laboratory tests, including blood cell counts, serum biochemistry and serum protein electrophoresis, were performed, and the clinical and laboratory alterations had reversed to normal.

**Fig. 1:**
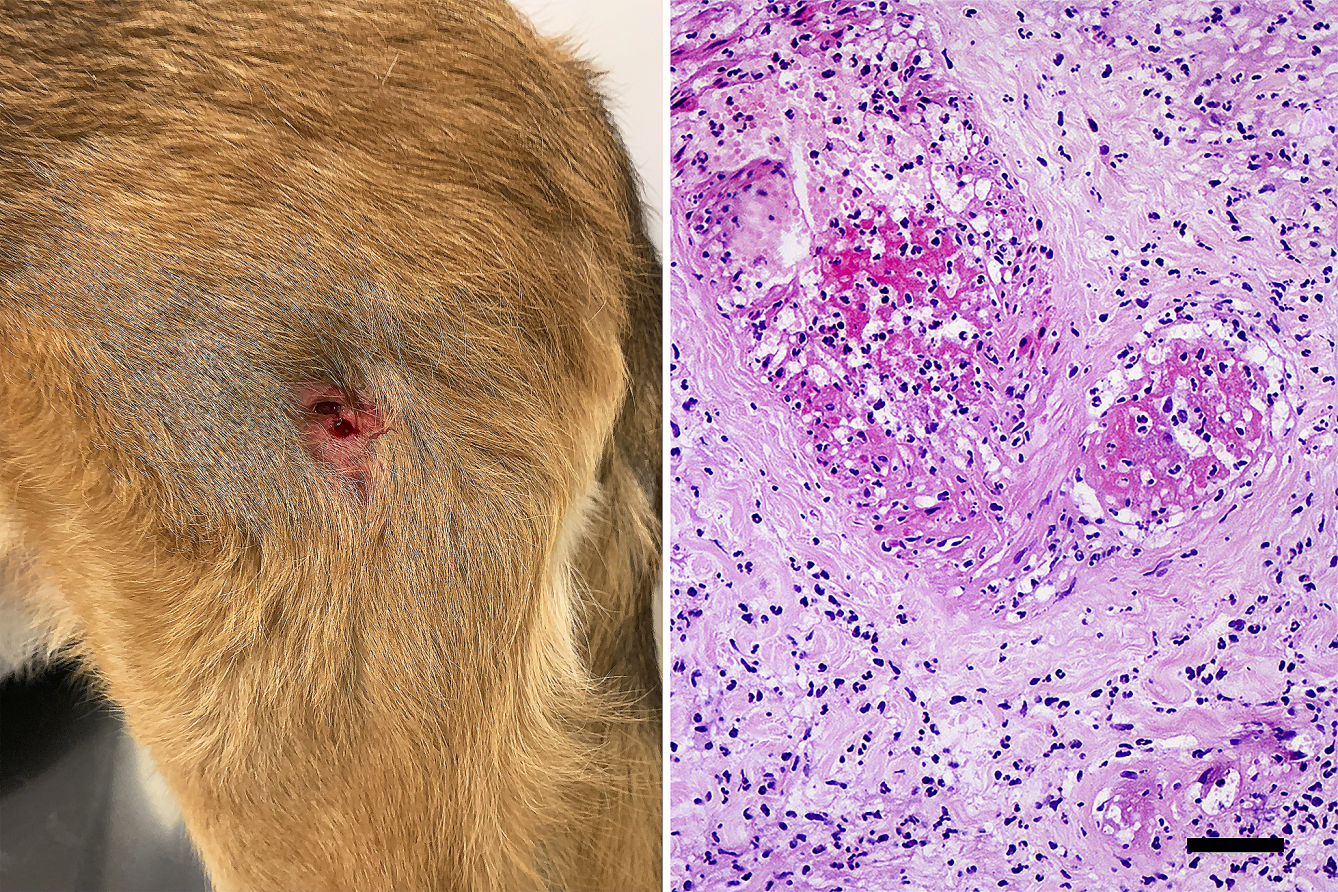
Cutaneous vasculitis, **a**). Focal ulcer on the lateral aspect of the left thigh; **b**), The wall of dermal blood vessels is disrupted and expanded by moderate deposition of fibrin admixed with degenerate neutrophils and cellular and karyorrhectic debris (fibrinoid necrosis). HE, scale bar: 50 μm

At the time of referral, the animal presented with a bilateral cutaneous ulcer diagnosed as vasculitis in the presence of amastigotes by histology and specific immunohistochemistry in the lateral aspect of the thigh and renal disease due to *L. infantum* infection (Fig. 1a). Haematology and biochemistry analyses were performed and revealed mild nonregenerative anaemia (Erci: 5.11 M/µL, reference interval (RI): 5.65–8.87), together with increased serum concentrations of BUN (109 mg/dl, RI: 3–27 mg/dl), creatinine (2.3 mg/dl, RI: 0.5–1.4 mg/dl), phosphorus (10.1 mg/dl, RI: 1.6–8.1 mg/dl), alanine aminotransferase (1027 U/L, RI: 10–88 U/L), aspartate aminotransferase (185 U/L, RI: 10–85 U/L), alkaline phosphatase (303 U/L, RI: 30–120 U/L) and gamma-glutamyl transferase (71 U/L, RI: 1–12 U/L). Additionally, the urine protein/creatinine ratio was 3.56 (RI: <0.5), and the specific weight of the urine was 1.012. Serum protein electrophoresis was also performed by agarose gel electrophoresis (AGE) with a HYDRAGEL Kit, which revealed polyclonal gammopathy and a spike in the alpha-2 fraction. Quantitative serology based on an in-house ELISA technique was performed to detect the presence of anti-*Leishmania* antibodies, and the results were markedly positive (218%, RI: >200%) (Villanueva-Saz et al. 2022). Quantitative serology based on commercial ELISA was performed to detect the presence and presence of circulating immune complexes (CICs) (mg/L) in serum samples following the instructions of the manufacturer (Canine CIC ELISA Kit, BlueGene Biotech, China). The kit consists of a competitive enzyme immunoassay technique utilizing a polyclonal anti-CIC antibody and a CIC-horseradish Peroxidase (HRP) conjugate. The assay sample and buffer were incubated together with the CIC-HRP conjugate in precoated plates for one hour. After the incubation period, the wells were decanted and washed five times. The wells were then incubated with a substrate for the HRP enzyme. The product of the enzyme–substrate reaction forms a blue-coloured complex. Finally, a stop solution was added to stop the reaction, after which the solution becomes yellow. The intensity of the colour was measured spectrophotometrically at 450 nm using a microplate reader. The intensity of the colour is inversely proportional to the CIC concentration from serum samples, and the CIC-HRP conjugate competes for the anti-CIC antibody binding site. High levels of CICs were detected (200 mg/L).

Due to the patient’s worsening clinical signs associated with leishmaniosis, including the presence of anorexia and exfoliative dermatitis, a new anti-*Leishmania* treatment, including meglumine antimoniate (30 mg/kg BID subcutaneously) and allopurinol (10 mg/kg BID PO), was given since the first treatment a year prior. Moreover, supportive treatment with benazepril (0.5 mg/kg SID PO) and methylprednisolone (0.33 mg/kg SID PO) was administered based on the presence of high levels of circulating immune complexes. In this sense, use of glucocorticoids in ill dogs has beneficial medical effects, as described previously (Roura et al. 2021).

**Fig. 2:**
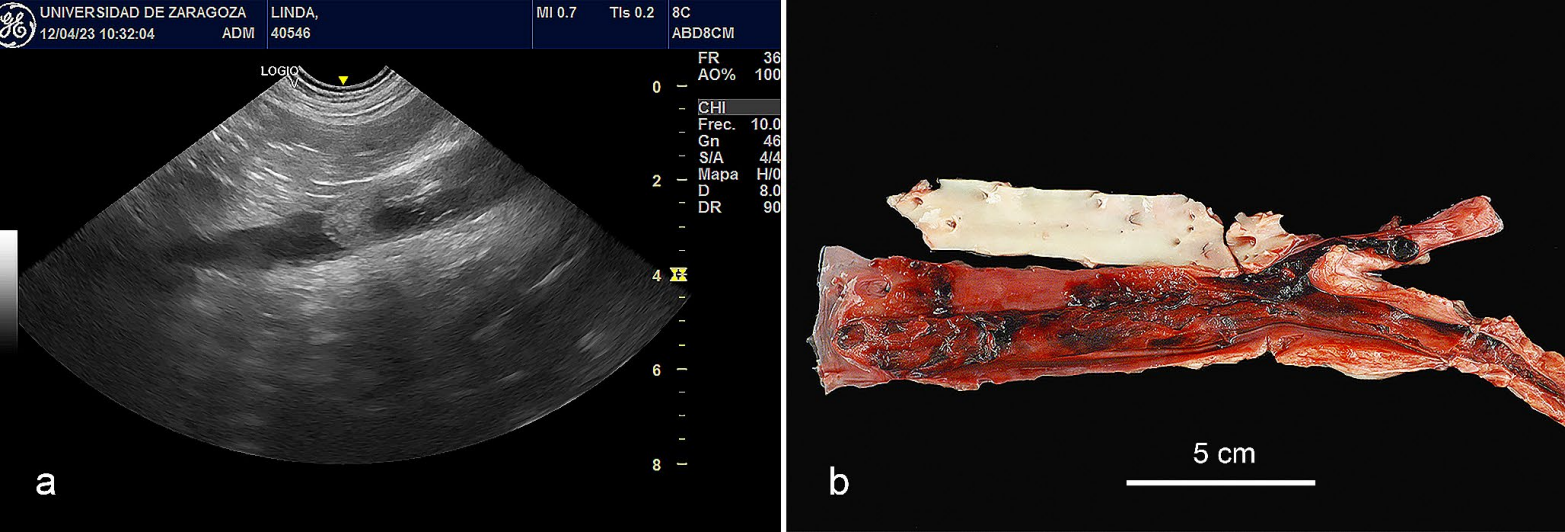
Large thrombi occupying the lumen of the vena cava and extending into the external iliac veins. **a**). Abdominal ultrasound showing an echogenic structure with focal areas of heterogeneity occupying the lumen of caudal vena cava and extending to the external iliac veins, not obtaining Doppler sign. This image was consistent with a thromboembolism of the caudal vena cava and external iliac veins. **b**). Reddish thrombus, of approximately 14 cm in length, occluding the lumen of the caudal vena cava and the external iliac veins

Two weeks after starting the second administration of meglumine antimoniate, the patient presented with acute claudication of the hind limbs. Complete blood analysis was performed, which showed a general worsening of the patient’s condition. Activated partial thromboplastin time (aPTT) and prothrombin time (PT) were measured, and in both cases, the values were within the normal range (aPTT = 92 s, RI = 72–102 s; PT = 16 s, RI = 11–17 s). Abdominal ultrasound revealed an echogenic structure with heterogeneous focal areas occupying the entire lumen of the caudal vena cava and the external iliac veins without Doppler signs (Supplementary Video 1). This image was consistent with thromboembolism (Fig. 2a). The animal was hospitalized, and a palliative analgesic treatment based on methadone (1 mg/kg SC) was established. The animal died naturally two hours later, and necropsy was authorized by the owners.

**Fig. 3:**
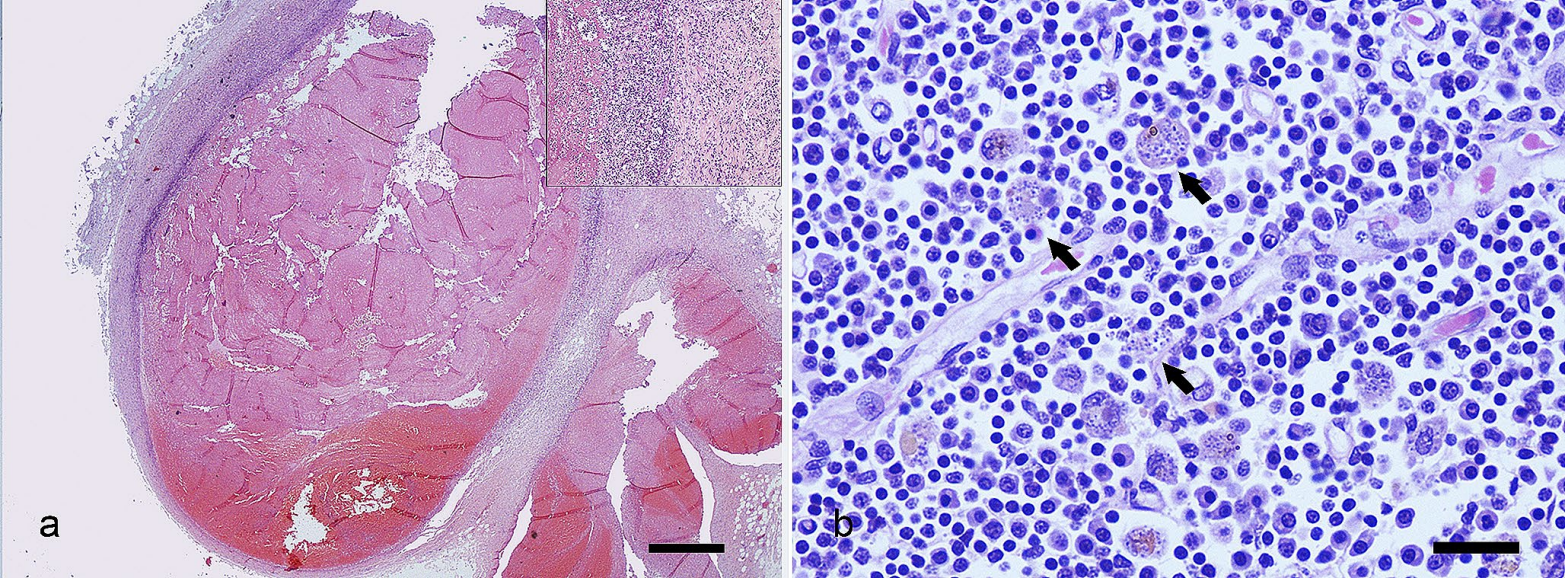
Transversal histologic sections of the caudal vena cava and mediastinal lymph node. **a).** Adhered to the intima of the caudal vena cava there is a large, organized thrombus. HE, scale bar: 1000 μm. **Inset**: The wall of the caudal vena cava is thickened and effaced by a transmural necrotizing and inflammatory process. HE. **b**). In lymph node, macrophages contains large numbers of oval microorganisms compatible with amastigotes of *Leishmania* spp (arrows). HE, scale bar: 20 μm

Postmortem examination revealed a large, reddish thrombus approximately 14 cm in length that occluded the lumen of the caudal vena cava and extended into the external iliac veins (Fig. 2b). Additionally, the renal cortex presented multiple infarcts. The affected tissues were fixed in 10% neutral-buffered formalin, embedded in paraffin, and cut into 4-µm thick sections, which were stained with haematoxylin and eosin (HE). Microscopically, the thrombus effaced the endothelium. The entire circumference of the vascular wall was thickened and obscured by a transmural necrotizing and inflammatory process composed mainly of viable and degenerated neutrophils, macrophages, and lymphocytes admixed with abundant fibrin and oedema (Fig. 3a). In the kidney, there was severe membrane-proliferative glomerulonephritis with occasional deposition of proteinaceous material in Bowman’s space and atrophy of renal glomeruli. The spleen and lymph nodes presented macrophages containing large numbers of oval microorganisms measuring approximately 3 to 4 μm in diameter, which is compatible with the morphology of *Leishmania spp*. (Fig. 3b).

To determine the presence of *Leishmania* parasites in the tissue sections, immunohistochemistry was performed using a standard protocol with an Autostainer Link48™ (Dako, Glostrup, Denmark) and an in-house rabbit polyclonal antibody specific for *L. infantum*. Blocking of endogenous peroxidase activity (Dako REAL™ Peroxidase-Blocking Solution, Dako, Glostrup, Denmark) was performed before the sections were incubated for 30 min with the primary antiserum at room temperature (RT) (1 in 500 dilution in EnVision Flex™ antibody diluent, Dako). Thereafter, the sections were incubated for 30 min at RT with Dako EnVision + System-HRP, Rb™. The substrate used for detection was 3.3′-diaminobenzidine, which was incubated for 10 min. The sections were then counterstained with haematoxylin for 8 min (EnVision™ FLEX Haematoxylin, Dako) and covered on slides. For the negative control, the primary antibody was replaced with nonimmune rabbit serum. A biopsy sample of the lymph node from a dog with clinical leishmaniosis and a dog negative for *L. infantum* infection were included as positive and negative controls, respectively. Immunohistochemistry revealed a positive signal within macrophages in the spleen and lymph nodes. However, the skin and affected veins were negative according to the specific immunohistochemistry.

## Discussion

The present report describes a case in which thrombophlebitis affecting the vena cava and external iliac veins occurred in a dog and potentially arose as a complication of *L. infantum* infection. The primary abnormalities that contribute to thrombosis include endothelial injury, stasis or turbulent blood flow, and hypercoagulability (commonly known as the Virchow triad) (Kumar et al. 2021a) In our case, thrombosis occurred without impacting coagulation parameters. Therefore, the probable cause of this thrombosis was associated with thrombophlebitis because of deposition of CICs. Although the pathogenesis of coagulopathies associated with *L. infantum i*nfection has not been fully elucidated, thrombophlebitis may have arisen due to the collective detrimental effects on haemostasis caused by *L. infantum* itself, coupled with hepatic and renal injuries. Furthermore, deposition of CICs within vessel walls can induce an inflammatory response, leading to vasculitis. Ideally, establishing a direct causal relationship between vasculitis and CIC deposition would require a direct immunofluorescence assay. Unfortunately, this technique is not widely available in most laboratories. Nevertheless, CIC levels were evaluated in the serum using a commercial ELISA, which revealed elevated CIC levels. This parameter correlated directly with anti-*Leishmania* antibody levels, as well as with the severity and progression of the disease (Parody et al. 2019). Given the numerous clinicopathological findings in CanL patients due to CIC deposition in various tissues, measuring CIC levels may serve as an indirect indicator of disease severity (Cacheiro-llaguno et al., 2021).

Clinical signs in dogs are caused by the presence of the parasite in a specific tissue and/or humoral immune response accompanied by the production of nonprotective anti-*Leishmania* antibodies, which generate large numbers of circulating immune complexes that deposit in basal membranes, possibly leading to polyarthritis, uveitis, glomerulonephritis, and vasculitis (Torrent et al. 2005; Solano-Gallego et al. 2011). Vasculitis in both human and canine leishmaniosis is mediated by a type III hypersensitivity reaction triggered by immunocomplex deposition (Torrent et al. 2005). When antibodies deposit in fixed tissues, injury can occur due to inflammation. The deposited antibodies activate complement, generating cleavage products, including chemotactic agents, which direct the migration of granulocytes and monocytes. Leukocytes are activated by engagement of their C3b and Fc receptors (Kumar et al. 2021b). In this patient, despite medical treatment, the high blood concentration of circulating anti-*Leishmania* antibodies led to the formation and deposition of immune complexes in vascular walls, triggering the pathogenesis described above.

In dogs with clinical leishmaniosis, vasculitis of the large blood vessels is not one of the most common clinical manifestations. These lesions are usually located on the margins of the ears and tip of the tail (Koutinas and Koutinas 2014). In our case, the presence of previous vasculitis in an atypical location with exaggerated immunocomplex formation and deposition in different tissues, including vessels, might have predisposed the patient towards thromboembolism formation.

In principle, deficiencies, or inhibitors of clotting factors within the extrinsic and final common pathways and in the intrinsic and common pathways, lead to prolongation of prothrombin time and activated partial thromboplastin time, respectively (Kamal et al. 2007). However, aPTT and PT were within normal limits on the day before the present patient died. Specifically, routine coagulation tests such as PT and aPTT are not useful for predicting the risk of thrombosis, as they evaluate only the time to the start of clot formation (Lim et al. 2022). For this reason and considering the poor prognosis that this clinical picture entails, it would be advisable to perform regular monitoring of the coagulation profile in *Leishmania*-infected dogs, especially in those developing kidney disease with nephrotic syndrome. A more global assessment of the coagulation cascade, including measurement of the final products of coagulation, such as fibrin and thrombin, would be necessary to ensure haemostatic function.

Additionally, the level of antithrombin, which is primarily produced in the liver, may decrease due to liver dysfunction, leading to a substantial risk of thrombosis (Toulza et al. 2006). Severe and chronic liver damage can induce various disruptions in coagulation, resulting in compensatory pro- and anticoagulant conditions and rebalanced haemostasis. However, this equilibrium is delicate and, in addition to being influenced by concurrent pathologies (such as kidney disease, vascular damage, or systemic inflammatory conditions), tends to naturally shift towards a procoagulant state.

Treatment for *Leishmania* involves administering meglumine antimoniate in combination with allopurinol, following the LeishVet guidelines for practical management of CanL (Solano-Gallego et al. 2011). Additionally, introduction of glucocorticoid therapy has recently been described as potentially beneficial. This therapy aims to reduce formation of circulating immune complexes (CICs) and mitigate the resulting organ damage (Roura et al. 2021).

In humans, hypercortisolism, including an increase in different coagulation factors and platelet hyperactivity, has been associated with increased thromboembolic risk (Wagner et al. 2019). For dogs, limited information is available related to thromboembolic risk. There is evidence that oral administration of glucocorticoids can cause a significant increase in endogenous thrombin potential, resulting in a hypercoagulability state detected by thrombelastography, but the effect on thrombin generation varies among dogs (Rose et al. 2011). Other studies have reported different results, with a significant decrease in antithrombin levels following use of anti-inflammatory and immunosuppressive agents after 15 days of treatment. However, an increase in platelet aggregation was observed in dogs receiving immunosuppressive doses of prednisone (Romão et al. 2013). In this sense, it is very difficult to correlate hypercortisolism due to corticosteroid therapy or endocrine disease with thromboembolism in dogs because the studies that evaluated these effects are vastly based on data from human studies (Romão et al. 2013).

A potential limitation is the impossibility of performing specialized staining techniques to detect deposition of circulating immune complexes, along with other methods such as thromboelastography and coagulation screening assays, such as D-dimer levels. Some of these techniques are not routinely available in veterinary clinical laboratories.

In summary, CanL is a rare complication in dogs that can present as acute hind-limb claudication resulting from thrombophlebitis affecting the caudal vena cava and external iliac veins. Determining the exact role of *L. infantum* infection in the development of thrombophlebitis is challenging due to limited access to certain diagnostic techniques and the presence of concurrent pathologies. Although this clinical presentation is reportedly uncommon, it underscores the importance of considering the impact of *L. infantum* infection on haemostasis, particularly in dogs with advanced disease and concurrent pathologies.

### Electronic supplementary material

Below is the link to the electronic supplementary material.

Supplementary Material 1

## Data Availability

The data that support the findings of this study are available from the corresponding author upon reasonable request.
